# Clinicoepidemiological Profile of Retinopathy of Prematurity

**DOI:** 10.7759/cureus.69831

**Published:** 2024-09-21

**Authors:** Sasmita Sahu, Sharmistha Behera, Gurudev Bishi, Lihalin Choudhury, Smruti Mishra

**Affiliations:** 1 Department of Ophthalmology, Veer Surendra Sai Institute of Medical Sciences and Research, Burla, Sambalpur, IND

**Keywords:** development, premature, retinopathy of prematurity, retinopathy screening, rop

## Abstract

Purpose

In previously performed studies, retinopathy of prematurity (ROP) has been shown to occur postpartum. As a result, mechanical ventilation and oxygen dependence were eventually connected to the development of ROP in these preterm children, and ROP prevalence is on the rise globally. Despite various improvements in childcare, ROP still tends to arise due to various risk factors associated with the disease. So, clinically the research was performed to determine the clinical and epidemiological profile of ROP.

Materials and methods

A total of 268 participants were to be enrolled in the study. It was an observational, cross-sectional study carried out at Department of Ophthalmology and Neonatal Intensive Care Unit in Veer Surendra Sai Institute of Medical Sciences and Research Centre, Burla, Sambalpur, Odisha, India. Ethical approval was provided on 30 November 2019.

Results

Overall 123 (46%) infants out of 268 infants developed retinopathy of prematurity. Majorly, the infants had stage 1 and stage 2 ROP. Most of the infants were male. Birth weight in ROP patients was 1000 g or less in 54% of infants, 1000-1500 g in 46% of infants and >1500 g in 33% of infants. Gestational age was found to be a predictor of retinopathy of prematurity. The adverse events that were found to be associated with retinopathy of prematurity were sepsis (53%), respiratory distress syndrome (RDS) (54.6%), intraventricular haemorrhage (IVH) (60%), anaemia (61.5%).

Conclusion

It has been observed that babies with higher birth weights and older gestations are also susceptible to developing ROP. Therefore, the greater birth weight newborns up to 1750 g and older gestational age babies >34 weeks should also be included in the screening criteria, especially if they have risk factors such as oxygen supplementation, sepsis, RDS, and anaemia.

## Introduction

Retinopathy of prematurity (ROP), formerly known as retrolental fibroplasia (RLF), was a prolonged disorder in the 20th century. The contemporary history of the disease's discovery was due to the scar tissue behind the lens of a newborn that linked it further to the retinal detachment. The mid-1950s and late 1970s witnessed epidemics of this ailment, which were spaced around 25 years apart [1].

The term retrolental fibroplasia was created after more instances were reported [2,3]. It became widespread in specialised facilities for preterm newborns worldwide, and a wealth of research on RLF and ROP has subsequently appeared [4,5]. It indicates that during the late 1940s to the mid-1950s, there was an "oxygen epidemic." During this time, ROP was the most common cause of newborn blindness [5-7]. In 1942, Terry described the first baby who had membranes behind the pupil coated in grey blood vessels [2,6,8].

It was realized that ROP developed after birth, and over time, causation in these preterm infants became linked to artificial ventilation and oxygen dependency. Over the ensuing 25 years, several elements such as sepsis, maternal factors, gestational age, poor nutrition, complications of pregnancy, phototoxicity, ischaemia, elevated oxygen level, low oxygen level, and iron deficiency were advanced as possible causes of the disease. However, the illness is becoming more common even with careful attention to oxygen use. In actuality, it has happened to both hypoxic and term babies who have never received extra oxygen. Consequently, the cause of this fibrovascular proliferation illness in the newborn retina is still mostly unknown [8].

ROP is known to be a preventable reason for blindness and vision-related morbidity. Further, many more very low birth weight (VLBW) and extremely low birth weight (ELBW) newborns who are at risk of developing ROP are surviving because of different technologies and newer advancements in neonatal intensive care units and neonatal health care facilities.

Thus, there is a global trend towards an increase in ROP incidence. To date, no good epidemiological data on ROP in Odisha is available. Hence, this study was performed to determine the outcomes of the clinical and epidemiological profile of ROP.

## Materials and methods

Study design

It was an observational, cross-sectional study. The study was conducted for two years, i.e., from October 2019 to September 2021. The study was performed at the Department of Ophthalmology and Neonatal Intensive Care Unit in Veer Surendra Sai Institute of Medical Sciences and Research Centre, Burla, Sambalpur, Odisha, India.

Study population

A total of 268 patients were enrolled in the study. The inclusion criteria of participants were <34 weeks of gestation, weight of child at birth <1750 g, with or without associated risk factors like sepsis, respiratory distress syndrome, and supplemental oxygen therapy needed. The exclusion criteria were term infants and intrauterine growth restriction (IUGR) infants.

Ethical approval

The study has been approved by the Institutional Research and Ethics Committee, Veer Surendra Sai Institute of Medical Sciences and Research Centre, Burla, Sambalpur, Odisha, India dated 30 November 2019 (approval 19215/Dt.30.11.19/IST-228/19).

Data Collection

The variables recorded in the duration of the study included gestational age in weeks, sex, birth weight in grams, and duration of oxygen therapy in days.

Study procedure

The obstetrical history and postnatal course were obtained from records. All newborns weighing less than 1750 g at birth and less than 34 weeks gestation were admitted to the neonatal intensive care unit and retina clinic after their parents gave their written consent in advance and based on the inclusion criteria. Ethical clearance for the study was obtained from the institutional ethics committee of the hospital. Preterm infants who met the eligibility requirements were registered. The risk factors for neonates included delivery method, septicemia, anaemia, respiratory distress syndrome (RDS), preterm, and intraventricular haemorrhages. Treatment specifics, such as the administration of blood products and oxygen treatment, were documented. Topical tropicamide (0.8%) at half strength and phenylephrine (5%) were instilled into both eyes until complete pupillary dilatation was achieved as this concentration is already considered for use in case of pupillary dilation. Proparaciaine drops, a topical anaesthetic, were instilled. Babies with positive results were taken to the ophthalmology outpatient department (OPD) so that their records may be kept in a retina camera (RetCam). The screening was conducted using RetCam, an indirect ophthalmoscope, and +20 lenses in the ophthalmology department, while +20 D lens and an indirect ophthalmoscope were used for screening in the newborn ICU. Retinopathy was classified according to the International Classification of Retinopathy of Prematurity (ICROP) into zones and stages. Infants who have normal vascularization up to the periphery of their retina were not re-examined. Infants who required treatment were then referred to higher centre where ROP treatment facilities were available.

Statistical analysis

Statistical analysis was done by recording, categorization, and computation with the help of Microsoft Excel (Redmond, WA, USA). Data has been presented as either n or n (%).

## Results

**Figure 1:**
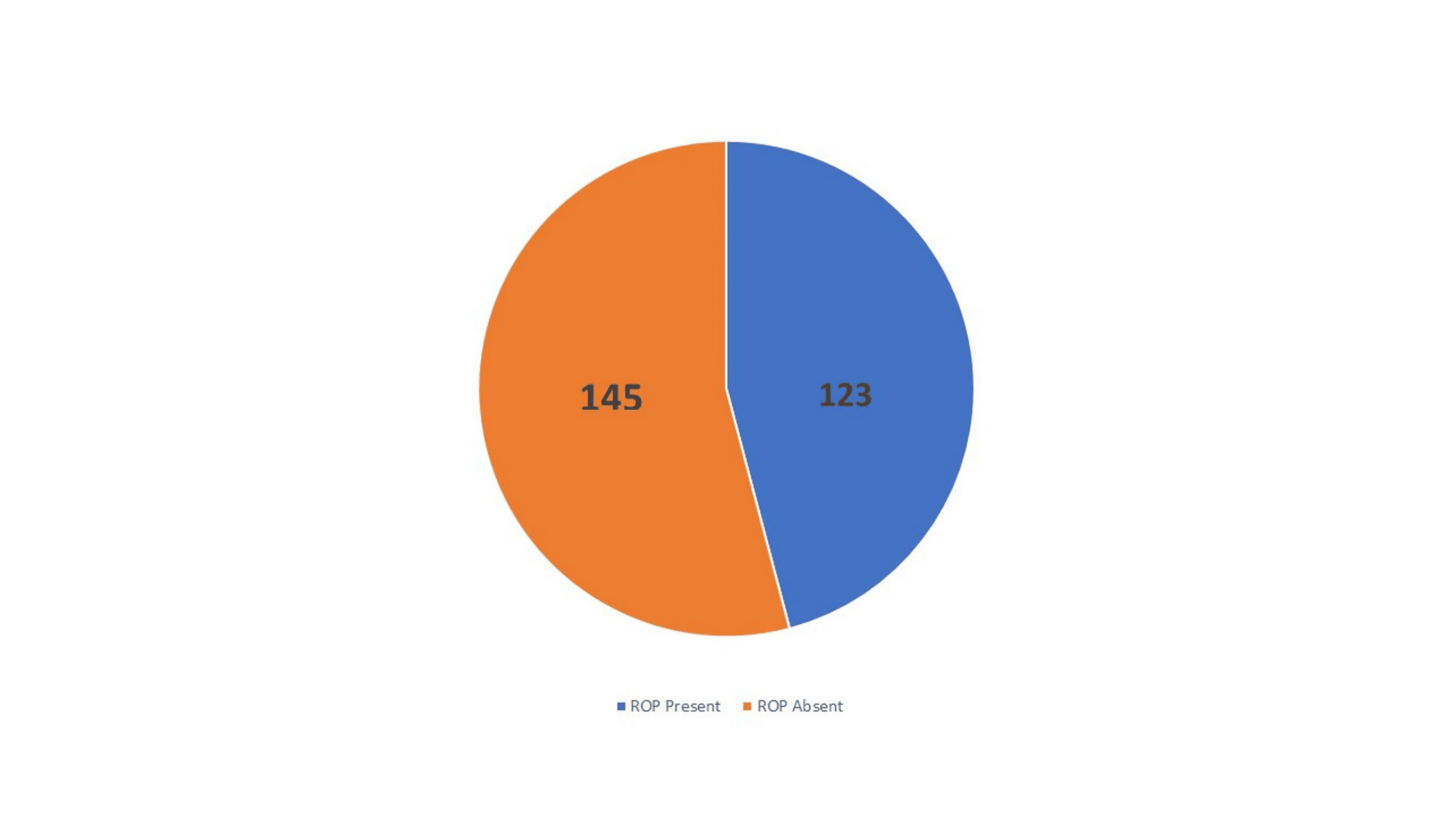
Proportion of retinopathy of prematurity (ROP) in infants

The study screened a total of 268 newborn babies with birth weight <1750 gm and/or gestational age <34 weeks. Out of 268 infants screened, only 123 of them developed ROP. The overall proportion of ROP was found to be 46%. Figure 1 depicts the proportion of retinopathy of prematurity in infants.

Table 1 represents study characteristics of participants. Out of 154 males 76 (49%) and out of 114 females 47 (41%) developed ROP. Out of the 126 newborns that were in the 26-30 week gestational age (GA) group, 73 (58%) had ROP and 50 (35%) of the 142 newborns that were in the >30-34 week GA group also had ROP. Similarly, of the 177 infants who had received supplemental oxygen, 102 (57.6%) developed ROP while 21 out of 91 (23%) who did not receive supplemental oxygen developed ROP.

**Table 1:** Study characteristics Data is presented as n (%) ROP: Retinopathy of Prematurity; GA: Gestational Age

	ROP Absent	ROP Present
Male	78 (51%)	76 (49%)
Female	67 (59%)	47 (41%)
GA 26-30 (in weeks)	53(42%)	73(58%)
GA>30-34 (in weeks)	92(65%)	50(35%)
Birth weight		
1000gm/less	28(46%)	33(54%)
>1000-1500gm	89(54%)	76(46%)
>1500gm	28(67%)	14(33%)
Oxygen Supplementation provided	75 (42.4%)	102 (57.6%)
Oxygen Supplementation not provided	70 (77%)	21(23%)

Table 2 represents various stages of retinopathy of prematurity. Of the 123 infants who were found to have ROP, 38 (31%) neonates were in stage 1, 38 (31%) in stage 2, 32 (26%) in stage 3, one (0.8%) in stage 4A, 13 (10.5%) in aggressive posterior retinopathy of prematurity (APROP) and one (0.8%) had total retinal detachment. Sixty-two percent of infants were in stage 1 and 2 and 26% were in stage 3 while 12% were in stage 4 or more and APROP.

**Table 2:** Stages of ROP Data is presented as n (%) ROP: Retinopathy of Prematurity; APROP: Aggressive Posterior Retinopathy of Prematurity; TRD: Tractional Retinal Detachment

STAGE	ROP Present
1	38 (31%)
2	38 (31%)
3	32 (26%)
4A	1 (0.8%)
APROP	13 (10.5%)
TRD	1 (0.8%)

Table 3 represents a few adverse outcomes that were present such as sepsis in 190 infants, RDS in 196 infants, Intraventricular haemorrhage (IVH) in 77 infants and anaemia in 13 infants.

Of the 190 infants who had sepsis 101 (53%) developed ROP whereas 22 out of 78 (28%) developed ROP. High significant correlation between sepsis and ROP is present and 196 infants who had RDS present 107 (54.6%) developed ROP while of the 72 infants who did not have RDS 16 (22.2%) developed ROP. Out of those 77 infants who had IVH, 46 (60%) developed ROP whereas 77 out of 191 (40%) who did not have IVH developed ROP and of 13 infants with anaemia eight (61.5%) developed ROP whereas out of 255 infants without anaemia 115 (45%) developed ROP. The statistically significant differences were observed in events like sepsis (p-value=0.0001) and RDS (p-value=0.002). The p-value for IVH was 0.08 and anaemia was 0.13, which was considered insignificant. 

**Table 3:** Safety outcomes Data is presented as n (%) ROP: Retinopathy of Prematurity

	ROP Absent	ROP Present
Sepsis	89 (47%)	101(53%)
Respiratory Distress Syndrome (RDS)	89 (45.4%)	107 (54.6%)
Intraventricular Haemorrhage (IVH)	31 (40%)	46 (60%)
Anaemia	5 (38.5%)	8 (61.5%)

## Discussion

Our study included 268 infants after reviewing medical records. Retinopathy of prematurity was identified in 123 newborns who weighed less than 1750 g at birth or had a gestational age of less than 34 weeks. The study with similar screening criteria conducted by Rekha and Battu showed an incidence of 46% ROP [9]. According to several Indian studies, the prevalence of ROP ranges from 20% to 60.2% [9]. Most of the infants enrolled in the study were males as compared to the females.

The findings of the study demonstrated that the majority of newborns without ROP belonged to the higher GA group, whereas the percentage of newborns with ROP was greater in the lower GA group. According to research by Vinekar et al. comparing Indian and Western youngsters, Indian newborns with bigger birth weights and later gestations had a higher risk of developing ROP than their counterparts in Western nations [10]. Similarly, Jalali et al. recommended that infants weighing less than 2000 g at birth and delivered at a gestational age of fewer than 37 weeks should likewise be tested [11]. However, in our study, it has been observed that the development of ROP with respect to the various birth weight groups are as follows: out of 61 infants of 1000 g or less 33 (54%), 165 infants of >1000 g to 1500 g (46%) and of 42 infants of >1500 g 14 (33%) developed ROP. Thus, it suggests that the lower the birth weight, greater are the chances of developing ROP. Additionally, a multicenter cryotherapy experiment revealed that the chance of developing ROP increases with decreasing birth weight [12]. Maheshwari et al. defined various screening criteria for ROP screening such as infants with a birth weight under 1500 g and/or a gestational age of 35 weeks were screened and Jalali et al. suggested birth weight <2000 g and gestational age <37 weeks for screening of ROP [11,13]. Also, studies done by Flynn et al. and Chaudhury et al. have confirmed the association between oxygen supplements and ROP [14,15].

Mainly, infants with positive retinopathy of prematurity of our study were classified in the stage 1 and stage 2. Others were present in stage 3, stage 4A, aggressive posterior retinopathy of prematurity and tractional retinal detachment. In another investigation, Kumar et al. found that the incidence of severe ROP was 4.7% [16] while Vinekar et al. reported 3.5% of cases of severe ROP required therapy [10]. On another aspect, higher incidences of severe ROP have been recorded by Ahuja et al. and Hungi et al., at 10.2% and 13.2%, respectively [17,18].

The adverse events occurred during the study were sepsis, RDS, IVH, and anaemia. All the events assessed were found a bit high in infants with retinopathy. Thus, studies by Vinekar et al. and Aggarwal et al. found that septicaemia was a significant risk factor [10,16].

The main limitation of the study was screening of such participants as it possesses a significant challenge especially in a country like India due to shortage of workforce. Another limitation was access to the tools that were used for the procedure to assess retinopathy of prematurity.

## Conclusions

The study clearly showed the importance of screening for retinopathy of prematurity in newborns. It concluded that early detection of at-risk newborns can effectively prevent the occurrence of retinopathy of prematurity. Babies with higher birth weights and older gestations are also susceptible to developing ROP. Therefore, in further upcoming research, the greater birth weight newborns and older gestational age babies should be included in the screening criteria, especially if they have risk factors such as oxygen supplementation, sepsis, respiratory distress syndrome, intraventricular haemorrhage and anaemia. As in staging of retinopathy of prematurity, tractional retinal detachment was found to be least among all stages.

## References

[1] Shah PK, Prabhu V, Karandikar SS, Ranjan R, Narendran V, Kalpana N (2016). Retinopathy of prematurity: past, present and future. World J Clin Pediatr.

[2] Terry TL (2018). Extreme prematurity and fibroblastic overgrowth of persistent vascular sheath behind each crystalline lens: I. Preliminary report. Am J Ophthalmol.

[3] Terry TL (1942). Fibroblastic overgrowth of persistent tunica vasculosa lentis in infants born prematurely: II. report of cases-clinical aspects. Trans Am Ophthalmol Soc.

[4] Kalina RE, Karr DJ (1982). Retrolental fibroplasia: experience over two decades in one institution. Ophthalmology.

[5] Patz A (1954). Oxygen studies in retrolental fibroplasia. Am J Ophthalmol.

[6] Terry TL (1945). Retrolental fibroplasia in premature infants. Arch Ophthalmol.

[7] Owens WC, Owens EU (1949). Retrolental fibroplasia in premature infants. Am J Ophthalmol.

[8] Hakeem AH, Mohamed GB, Othman MF (2012). Retinopathy of prematurity: a study of prevalence and risk factors. Middle East Afr J Ophthalmol.

[9] Rekha S, Battu RR (1996). Retinopathy of prematurity: incidence and risk factors. Indian Pediatr.

[10] Vinekar A, Dogra MR, Sangtam T, Narang A, Gupta A (2007). Retinopathy of prematurity in Asian Indian babies weighing greater than 1250 grams at birth: ten year data from a tertiary care center in a developing country. Indian J Ophthalmol.

[11] Jalali S, Anand R, Kumar H, Dogra MR, Azad R, Gopal L (2003). Programme planning and screening strategy in retinopathy of prematurity. Indian J Ophthalmol.

[12] Palmer EA, Flynn JT, Hardy RJ (1991). Incidence and early course of retinopathy of prematurity. Ophthalmology.

[13] Maheshwari R, Kumar H, Paul VK, Singh M, Deorari AK, Tiwari HK (1996). Incidence and risk factors of retinopathy of prematurity in a tertiary care newborn unit in New Delhi. Natl Med J India.

[14] Flynn JT, Bancalari E, Bawol R (1987). Retinopathy of prematurity. Ophthalmology.

[15] Gopal L, Sharma T, Ramachandran S, Shanmugasundaram R, Asha V (1995). Retinopathy of prematurity: a study. Indian J Ophthalmol.

[16] Kumar P, Sankar MJ, Deorari A, Azad R, Chandra P, Agarwal R, Paul V (2011). Risk factors for severe retinopathy of prematurity in preterm low birth weight neonates. Indian J Pediatr.

[17] Hungi B, Vinekar A, Datti N (2012). Retinopathy of prematurity in a rural neonatal intensive care unit in South India-a prospective study. Indian J Pediatr.

[18] Ahuja AA, V Reddy YC, Adenuga OO, Kewlani D, Ravindran M, Ramakrishnan R (2018). Risk factors for retinopathy of prematurity in a district in South India: a prospective cohort study. Oman J Ophthalmol.

